# Impact of deep learning image reconstruction on volumetric accuracy and image quality of pulmonary nodules with different morphologies in low-dose CT

**DOI:** 10.1186/s40644-024-00703-w

**Published:** 2024-05-09

**Authors:** L. D’hondt, C. Franck, P-J. Kellens, F. Zanca, D. Buytaert, A. Van Hoyweghen, H. El Addouli, K. Carpentier, M. Niekel, M. Spinhoven, K. Bacher, A. Snoeckx

**Affiliations:** 1 Department of Human structure and repair, Faculty of Medicine and Health Sciences, Ghent University, Proeftuinstraat 86, 9000 Ghent, Belgium; 2 Faculty of Medicine, University of Antwerp, Universiteitsplein 1, Wilrijk, Belgium; 3 Department of Radiology, Antwerp University Hospital, Drie Eikenstraat 655, Edegem, Belgium; 4 Center of Medical Physics in Radiology, Leuven University, University Hospitals Leuven, Herestraat 49, Leuven, Belgium; 5 Cardiovascular Research Center, OLV Ziekenhuis Aalst, Moorselbaan 164, Aalst, Belgium

**Keywords:** Computed tomography, Deep learning image reconstruction, Iterative reconstruction, Lung cancer screening, Nodule volumetry, Nodule morphology, Image quality, Anthropomorphic chest phantom

## Abstract

**Background:**

This study systematically compares the impact of innovative deep learning image reconstruction (DLIR, TrueFidelity) to conventionally used iterative reconstruction (IR) on nodule volumetry and subjective image quality (IQ) at highly reduced radiation doses. This is essential in the context of low-dose CT lung cancer screening where accurate volumetry and characterization of pulmonary nodules in repeated CT scanning are indispensable.

**Materials and methods:**

A standardized CT dataset was established using an anthropomorphic chest phantom (Lungman, Kyoto Kaguku Inc., Kyoto, Japan) containing a set of 3D-printed lung nodules including six diameters (4 to 9 mm) and three morphology classes (lobular, spiculated, smooth), with an established ground truth. Images were acquired at varying radiation doses (6.04, 3.03, 1.54, 0.77, 0.41 and 0.20 mGy) and reconstructed with combinations of reconstruction kernels (soft and hard kernel) and reconstruction algorithms (ASIR-V and DLIR at low, medium and high strength). Semi-automatic volumetry measurements and subjective image quality scores recorded by five radiologists were analyzed with multiple linear regression and mixed-effect ordinal logistic regression models.

**Results:**

Volumetric errors of nodules imaged with DLIR are up to 50% lower compared to ASIR-V, especially at radiation doses below 1 mGy and when reconstructed with a hard kernel. Also, across all nodule diameters and morphologies, volumetric errors are commonly lower with DLIR. Furthermore, DLIR renders higher subjective IQ, especially at the sub-mGy doses. Radiologists were up to nine times more likely to score the highest IQ-score to these images compared to those reconstructed with ASIR-V. Lung nodules with irregular margins and small diameters also had an increased likelihood (up to five times more likely) to be ascribed the best IQ scores when reconstructed with DLIR.

**Conclusion:**

We observed that DLIR performs as good as or even outperforms conventionally used reconstruction algorithms in terms of volumetric accuracy and subjective IQ of nodules in an anthropomorphic chest phantom. As such, DLIR potentially allows to lower the radiation dose to participants of lung cancer screening without compromising accurate measurement and characterization of lung nodules.

**Supplementary Information:**

The online version contains supplementary material available at 10.1186/s40644-024-00703-w.

## Introduction

According to the 2020 Global Cancer Statistics, lung cancer remains the second most commonly diagnosed cancer and the leading cause of cancer-related death [1]. Mortality rates are high, as lung cancer is often diagnosed at an advanced stage when cure is no longer possible. Over the last decade, two large randomized controlled trials have demonstrated that lung cancer specific mortality is significantly lower among high risk participants who underwent screening with low-dose Computed Tomography (CT) compared to screening with chest radiography or no screening at all [2–4]. Consequently, thorough research on all aspects of lung cancer screening (LCS) has gained momentum in many European countries to prepare and support implementation of LCS on a national level [5–9].

Despite the benefits of LCS, it is of utmost importance that radiation risks of screening, like radiation-induced secondary cancers, are considered and that radiation doses are as low as reasonably achievable (ALARA principle) [10, 11]. Historically, many advancements have been made in CT technology driven by efforts to minimize the radiation dose. Image reconstruction techniques have been one of the main areas of development over the last decades to implement proper treatment of image noise in dose reduction techniques [12–15]. Compared to filter back projection (FBP), iterative reconstruction (IR) methods generally provide fewer artifacts and relatively higher signal-to-noise ratios for a given dose level [10, 15–19]. The hybrid IR methods, like ASIR-V (GE Healthcare), iteratively filter raw imaging data in combination with a backward projection, resulting in high reduction of artifacts and image noise [14]. Downside of these algorithms is that reconstruction times are long and that it produces images that appear blotchy, waxy- or plastic-looking which compromises detection of small lesions and nodules [12, 13]. This alteration in image texture, to which radiologists are generally less inclined, is caused by a shift in the noise power spectrum [13, 16]. Especially when reducing the radiation dose to the level of a chest radiography, this will affect the visibility of subtle image features and reduce object detectability [15, 20]. Nowadays with advancements in artificial intelligence (AI) and more readily available computing power, deep learning image reconstruction (DLIR) has gained more and more attention in the field of CT as it is able to generate high-quality images from low-dose sinogram input. DLIR is proposed as the solution in providing better image quality with desirable noise properties of FBP at acquisition doses and reconstruction times that outperform IR [11, 15, 17, 19, 21, 22]. Although the underlying working mechanism of these DLIR techniques is not fully know, these resulting denoising features appear to be particularly interesting in the context of low-dose CT screening.

LCS requires an adequate level of image quality as radiologists want to detect lung nodules when still small and characterize them as accurate as possible to provide appropriate work-up and/or follow-up management [3, 4]. This way, participants with an early diagnosed lung cancer have a better prognosis with improved five-year survival rates and expanded eligibility for curative surgical treatment [3, 23, 24]. Per definition, pulmonary nodules are round opacities in the lung that are well or poorly defined and measure up to 3 cm in diameter [25]. Even though there is a positive correlation between nodule size and malignancy, small nodules also have a likelihood of being malignant [23]. Nowadays, nodule management is primarily driven by nodule size, preferably determined by volumetry but diameter measurements are also possible [24, 26–28]. Therefore, accurate measurements of the size are essential. However, evaluation of benignity or malignity of nodules should not be based solely on size estimates. The LCS trials reported that up to half of detected lung cancers were adenocarcinomas, emphasizing the importance of investigating those morphologies resembling invasive, irregular forms of lung nodules [3, 4, 26]. Indeed, lobulated shapes and spiculated margins are features that are reported to be highly associated with a malignant nature [3, 23]. Lobulation arises when different parts within the nodule have uneven growth rates [23]. Spiculation is the radial and unbranched extension from the boundary of the nodule into the lung parenchyma [23]. Nodule management guidelines by the American College of Radiology (Lung-RADS 2022) or the British thoracic society (BTS guidelines) increasingly acknowledge the importance of morphology. The shapes and margins of pulmonary nodules should no longer be overlooked and should also be considered in synergy with size considerations in research [23, 26]. In the context of LCS, where a low radiation dose is a prerequisite, accurate characterization of all nodule characteristics must be preserved. However, CT image acquisitions with extreme dose reduction may impair the accuracy of volumetric assessment and the characterization of morphology because of increased noise levels [26].

As such, recent studies have investigated the role of DLIR in low-dose CT imaging by analyzing the objective and subjective image quality (IQ). Most of these studies on the one hand used physical evaluation phantoms to perform a technical assessment of IQ based on noise, noise power spectrum, task-based transfer functions, modulation transfer functions, detectability index, spatial resolution etc. [15, 17, 22, 29]. Those conventional quantitative metrics indicate that DLIR has the potential to generate images with objectively less noise. It is however important to keep in mind that those phantoms are far removed from real-life situations. As such, the improvements in objective IQ parameters from DLIR are not necessarily directly correlated with improvements in diagnostic accuracy [30]. On the other hand, another group of studies has used patient images to additionally investigate subjective scoring of IQ [30, 31]. The reported improved IQ of DLIR must however be put in perspective to the uncertainties related to the real-life patient set-up and their variabilities. For example, in most cases these patients were only scanned at one radiation dose, making it impossible to compare the same patient/ set-up at multiple dose levels [26, 30]. Besides, images could have been taken with or without contrast enhancement, where the intensity of the contrast agent in function of time could complicate direct pairwise comparison of contrast-to-noise ratios across different scans and reconstructions [32]. Between scans of different patients, variations in slice thickness have potential influence on noise and spatial resolution outcomes. Also, when CT scanners and reconstruction algorithms of different manufacturers are used it impedes pairwise comparisons. Lastly, within the same patient differences in lung volume/fill affect volumetry measurements.

Therefore, the purpose of this study was to investigate whether the nodule volumetric accuracy and subjective IQ perception of a new image reconstruction technique based on DL performs at least as good as the conventionally used IR algorithm. To this end, we established an anthropomorphic chest phantom CT dataset, resembling clinical daily practice. The exhaustive dataset allows to methodically investigate the impact of CT acquisition dose, reconstruction algorithm and reconstruction kernel on two metrics. These two metrics are semi-automatic volumetry measurements and subjective image quality, related to morphological nodule assessment.

## Materials and methods

### Anthropomorphic phantom

The multipurpose anthropomorphic chest phantom (Lungman phantom, Kyoto Kaguku Inc., Kyoto, Japan [33]) was used to acquire a standardized CT dataset. The phantom encloses an internal removable polyurethane structure, mimicking the pulmonary vessels and bronchi (up to the first bifurcation) connected to the mediastinum. These structures are three dimensionally dispersed in the phantom lung field that is naturally filled with air. Furthermore, synthetic bones of the chest made from epoxy resins are embedded in the phantom. To accommodate to a hypothetical screening situation of a European participant, the accompanying chest plates/ fat slabs (30 mm) were utilized during image acquisition (male, 82 kg, 168 cm, Body mass index of 29). The arms of the phantom are in abducted position, which further aligns with conventional positioning of patients and participants during chest CT-examinations.

### 3D-printed lung nodules

A set of 18 isolated nodule structures were 3D-printed in a material with a density of 1.17 g/cm^3^ (Resin Clear V4; Formlabs, Somerville, MA, USA) which appears radiodense in lung window and simulates solid lung nodules. The nodules can be subdivided in three morphology classes being lobular, spiculated and smooth. Per morphology class, nodules were printed with different diameters, starting from 4 to 9 mm with an increment of 1 mm. The nodules were randomly affixed between the pulmonary vessels of the Lungman phantom. For the determination of the clinically relevant reference volume of each of the 18 nodules, we calculated the average volume across the five radiologists measured on high dose CT scans (CTDI_vol_ 11 mGy) reconstructed with ASIR-V 60%. Table 1 summarizes the information of the pulmonary nodule set.

**Table 1:** Characteristics of the set of 18 3D-printed pulmonary nodules

***Nodule Diameter (mm)***	***Morphology type***	***Volume***_***High dose CT***_*** (mm***^***3***^***)***	***Standard Deviation (mm***^***3***^***)***
***4***	*Lobulated*	*29.0*	*0*
	*Spiculated*	*28.3*	*0.6*
	*Smooth*	*34.0*	*0*
***5***	*Lobulated*	*57.8*	*0.5*
	*Spiculated*	*51.5*	*1.0*
	*Smooth*	*68.3*	*0.5*
***6***	*Lobulated*	*99.3*	*3.5*
	*Spiculated*	*93.5*	*1.0*
	*Smooth*	*117.8*	*0.5*
***7***	*Lobulated*	*156.5*	*1.0*
	*Spiculated*	*144.5*	*3.0*
	*Smooth*	*188.5*	*1.0*
***8***	*Lobulated*	*244*	*4.0*
	*Spiculated*	*205.5*	*0.6*
	*Smooth*	*282.5*	*3.8*
***9***	*Lobulated*	*339.3*	*1.5*
	*Spiculated*	*300.8*	*0.5*
	*Smooth*	*391.3*	*1.5*

The 3D-printed nodules were affixed in the anthropomorphic chest phantom. All nodules have the same density (1.17 g/cm^3^). The column volume_High dose CT_ presents the ground truth reference volumes of the nodules determined as the average of five measurements on CT scans acquired at a CTDI_vol_ of 11 mGy, reconstructed with adaptive statistical iterative reconstruction at 60% blending (ASIR-V 60%). Standard deviations over the five measurements per nodule are presented in the last column

### Image acquisition and reconstruction

**Fig. 1:**
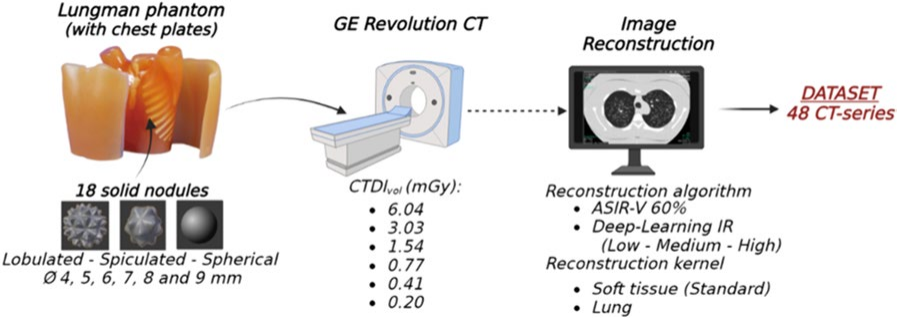
Schematic representation of how a standardized CT dataset was established. Abbreviations: CTDIvol: Volumetric Computed Tomography Dose Index, ASIR-V 60%: adaptive statistical iterative reconstruction at 60% blending, IR: image reconstruction

CT images of the Lungman phantom were acquired using the 256-slice GE Revolution CT scanner. Midline of the phantom was positioned in the isocenter. The CT scanner was operated at a tube voltage of 100 kVp, 40 mm collimation, pitch 0.98 and a gantry rotation time of 0.35 seconds. A total of six helical scan series were taken at different dose levels. The applied volumetric CT dose index (CTDI_vol_) values were 6.04 mGy (routine clinical chest protocol University Hospital Antwerp), 3.03, 1.54, 0.77, 0.41 and 0.20 mGy. In all cases tube current modulation (TCM) was used. The phantom, containing the 18 printed lung cancer nodules, remained at the same position to ensure that nodules are in the same place for each acquisition. Each of these six CT scans was reconstructed with a slice thickness of 1.25 mm and either a standard/soft tissue kernel or a hard/lung reconstruction kernel. The applied reconstruction algorithms included the routinely used volume adaptive statistical iterative reconstruction at 60% blending (ASIR-V 60%) and the TrueFidelity (GE Healthcare) DLIR at a low, medium and high level strength [34]. Schematic summary of the image acquisition of the anthropomorphic phantom together with the set of 18 nodules is depicted in Fig. 1.

### Nodule measurement and scoring

Five independent radiologists (experience ranging from 2 to 14 years) were asked to measure the volume and score the IQ of the 18 lung nodules on each of the 48 CT series. All image series were presented in a random order and blinded for dose and reconstruction parameters. Images were presented on a clinical PACS environment using a high-contrast color monitor (Barco MDCC-4430) at optimal lighting conditions.

### Nodule volumetry

All nodule volumes were determined using Lung VCAR semi-automated volumetry software (GE Healthcare) which is available in the PACS environment [35]. With this tool, radiologists manually initiate volumetry by providing a seed point to the software. The software then performs an automatic segmentation of the nodule and determines its volume in mm^3^. If the software tool was unable to give semi-automatic segmentation and volume determination, the radiologists were instructed to leave the form entry blank. Furthermore, they did not have to segment and correct segmentations and volumetry measurements manually. The individual volumetric measurements are then compared to the ground truth reference volumes depicted previously in Table 1. For this we used the clinically relevant reference volumes determined on the high dose (CTDI_vol_ 11 mGy) images. The absolute percentage volumetric error (APE_volume_) between individual measurements and ground truth values is then calculated with the formula depicted below.

$${APE }_{volume}\left(\%\right)=\left|\frac{Measured \,volume \left({mm}^{3}\right)-Ground \,truth \,volume \left({mm}^{3}\right)}{Ground \,truth \,volume \left({mm}^{3}\right)} \right|x 100$$

### Image quality score

Subjective IQ was interpreted for each of the 18 nodules on each of the 48 image acquisitions in a side-by-side comparison with the same high dose reference images as mentioned before (CTDI_vol_ 11 mGy). Radiologists recorded the perceived IQ as a score from 1 to 5 on an adapted five-point Likert scale, where nodules were perceived with a quality as good as on the high dose reference images (IQ score 5), minor reduction in quality compared to the high dose images (IQ score 4), moderate image quality (IQ score 3), very bad image quality (IQ score 2) or almost not visible in comparison to the high dose images (IQ score 1).

### Statistical analysis

Statistical analysis was performed in RStudio [36, 37] with the statistical software package MASS [38]. Graphs were generated with GraphPad Prism version 8.0.2 [39].

### Nodule volumetry

Via two multiple linear regression models, the influence of several predictor variables (radiation dose, reconstruction algorithm, reconstruction kernel, nodule morphology and diameter) was estimated on the outcome variable (calculated APE_volume_). The categorical predictor variables were included as dummy coded binary variables. Furthermore, the regression models included two-way interaction terms between the predictor variables. Assumptions for multiple linear regression were checked and the APE_volume_ data follows a log-normal distribution. Outlier identification was performed with the ROUT method. Since nodule volumetry was performed with the semi-automatic, observer-independent Lung VCAR tool without manual editing, we did not include random effects related to interreader variability. A first linear regression model was used to estimate volumetric errors of all nodules for the different doses, reconstruction algorithms and kernels. A second linear regression analysis was performed analogously to examine whether APE_volume_ varied when changing the predictor variables nodule diameter, morphology and reconstruction algorithm. To reduce multicollinearity between predictor variables, the continuous predictor variable radiation dose was standardized by subtracting the mean of each dose value and dividing the difference by the standard deviation of dose values. Output coefficients of both multiple linear regression models gave a general estimate of the absolute error that would be scored for a specific nodule in a particular image acquisition. Based on F-statistics, we can determine which predictor variables or interaction terms significantly influence the outcome variable APE_volume_. *P*-values (two-sided) smaller than 0.05 indicated a statistically significant impact.

### Image quality score

The ordinal, categorical IQ score data was analyzed with a mixed-effect ordinal logistic regression model. The binary outcome of the logistic regression model allows estimation of the possibility for a radiologist to allocate a particular IQ score (1 to 5) to a nodule on a certain image acquisition. As subjective image quality assessment intrinsically varies between different radiologists, the model was adapted to account for this via inclusion of random intercepts. Further, all main predictors as well as their interaction effects were included in the model, analogous to the volumetry analysis. From the output of the ordinal logistic regression we can calculate odds ratios that nodules with certain diameter and morphology are scored a particular IQ score. Based on likelihood ratio Chi square statistics, we determined which predictor variables or interactions terms have a significant effect on the perceived subjective IQ. *P*-values smaller than 0.05 indicated a statistically significant impact.

## Results

### Volumetric accuracy for varying radiation doses

Table 2 summarizes the predictor variables included in the first multiple linear regression analysis, their two-way interaction terms and strength with which they have an influence on the estimates of the APE_volume_ as determined by the F-statistics and p-values. It can be observed that all main predictor variables (dose, reconstruction algorithm and kernel) have a significant influence on volumetric accuracy. For the interaction effects, only the interaction between dose and reconstruction algorithm showed no significant effect. No variability was detected between different radiologists.

**Table 2:** Predictor and outcome variables of first multiple linear regression analysis with their according F-statistic

**Outcome variable**			
**APE**_**volume**_			
**Predictor variable (main effects)**	F-statistic	*p*-value	
Dose	12.60	3.67E-12***	
Reconstruction algorithm	49.69	< 2.2E-16***	
Reconstruction kernel	297.50	< 2.2E-16***	
**Predictor variable (interaction effects)**			
Dose – Reconstruction algorithm	0.69	0.80ns	
Dose – Reconstruction kernel	16.55	3.35E-16***	
Reconstruction algorithm – Reconstruction kernel	11.70	1.25E-07***	

Abbreviations: *APEvolume* Absolute percentage volumetric error. (Significance codes: ***p < 0.001, ** < 0.01, *p < 0.1, *ns* not significant)

**Fig. 2:**
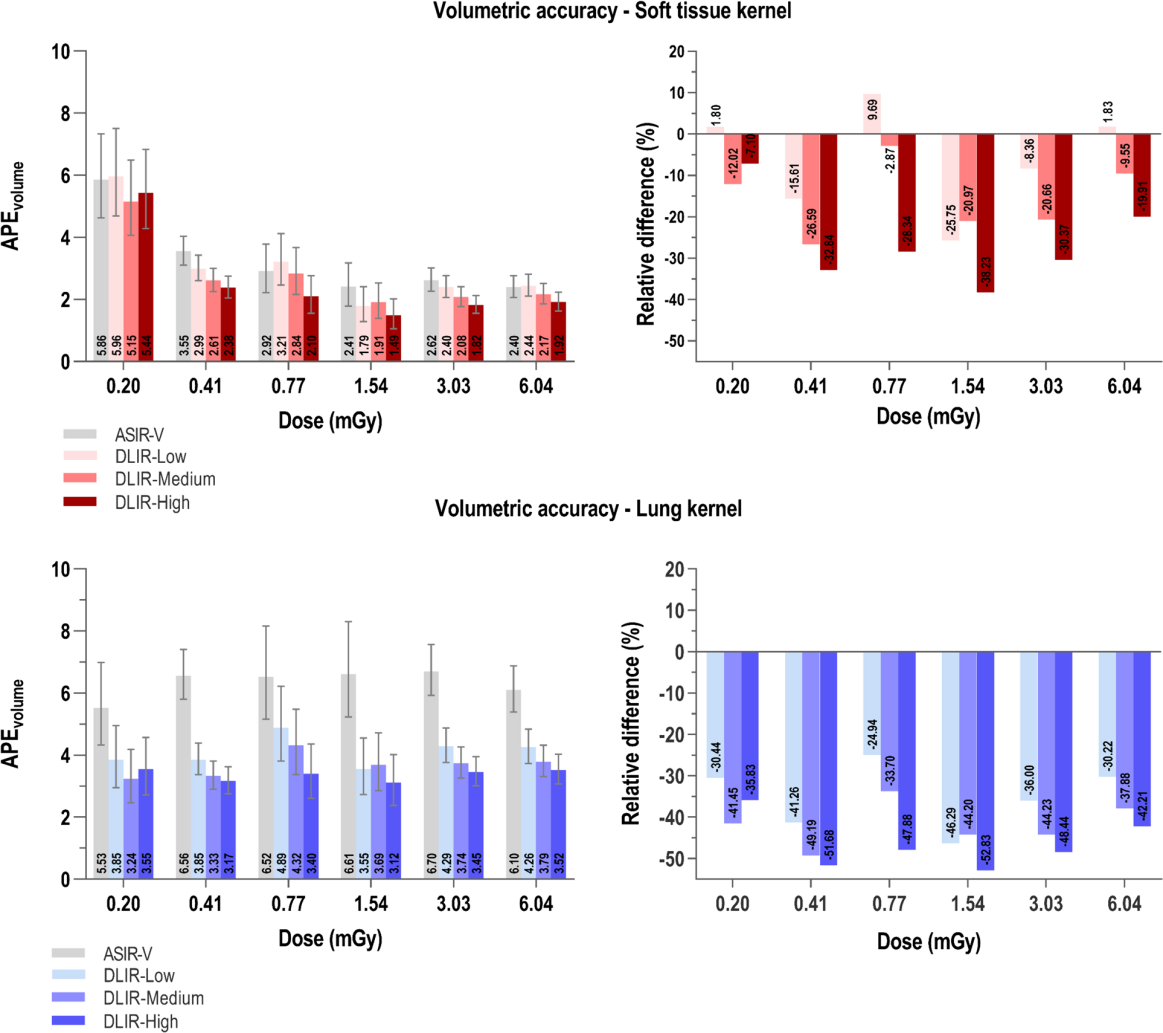
Volumetric accuracy in function of dose and grouped by reconstruction algorithm. Left: Absolute percentage volumetric error (APE_volume_) in function of radiation dose (mGy) for four different reconstruction algorithms (ASIR-V 60%, DLIR-Low, -Medium and -High) and subdivided for two reconstruction kernels (soft tissue and lung kernel). Error bars depict the 95% confidence intervals to display the variability on the estimated outcome. Right: Relative difference (%) in APE_volume_ of ASIR-V compared to different levels of DLIR in function of radiation dose, subdivided for two reconstruction kernels. Abbreviations: APE_volume_: Absolute percentage volumetric error, ASIR-V: adaptive statistical iterative reconstruction, DLIR: deep learning image reconstruction

Graphical representation of the impact of the four different reconstruction algorithms at the six radiation doses (in mGy) on volumetric accuracy can be found in Fig. 2 for both the soft tissue as well as the lung kernel. On the left side of Fig. 2, estimates of APE_volume_ derived by the linear regression model are shown. For the soft tissue reconstruction kernel there is an overall reduction in APE_volume_ for increasing radiation doses. With the lung kernel, APE_volume_ values remain mainly constant for the six different doses. When comparing the effect of reconstruction algorithms for each of the individual dose levels, it can be observed that DLIR generally, but not exclusively, showed lower estimates of volumetric error. For the soft tissue kernel DLIR-Low renders higher error estimates at 0.20, 0.77 and 6.04 mGy. At all dose levels for the lung kernel, ASIR-V renders higher APE_volume_ values than all levels of DLIR. Furthermore, a general and slight trend of volumetric error reduction can be observed when increasing the strength of DL, especially for the lung kernel.

Depicted on the right side of Fig. 2 are the relative differences (in percentage) in volumetric error between on the one hand ASIR-V and on the other hand three levels of DLIR. Negative values indicate that interchanging ASIR-V for DLIR (low, medium or high strength) renders lower volumetric errors. Accordingly, positive values show where ASIR-V allowed more accurate volumetric measurement than DLIR. The latter can be observed for the comparison between ASIR-V and DLIR-Low at 0.20, 0.77 and 6.04 mGy for the soft tissue kernel. In all other cases, interchanging ASIR-V for DLIR resulted in higher volumetric accuracy.

### Volumetric accuracy at standardized dose

Table 3 summarizes the predictor variables included in the multiple linear regression model, their two-way interaction terms and strength with which they have an influence on the estimates of the APE_volume_ as determined by the F-statistics and p-values. We split the model for the two different reconstruction kernels. All main predictor variables (reconstruction algorithm, morphology and diameter) as well as all their interactions have a significant influence on volumetric accuracy.

**Table 3:** Predictor and outcome variables of second multiple linear regression analysis with their according F-statistic

**Outcome variable**						
**APE**_**volume**_						
	**Soft tissue kernel**			**Lung kernel**		
**Predictor variable (main effects)**	F-statistic	*p*-value		F-statistic	*p*-value	
Reconstruction algorithm	15.77	3.9E-10***		76.74	< 2.2E-16***	
Nodule morphology	71.32	< 2.2E-16***		13.13	2.2E-06***	
Nodule diameter	56.29	< 2.2E-16***		66.09	< 2.2E-16***	
**Predictor variable (interaction effects)**						
Reconstruction algorithm – Nodule morphology	4.31	2.5E-04***		4.05	4.8E-04***	
Reconstruction algorithm – Nodule diameter	2.73	3.6E-04***		4.09	2.1E-07***	
Nodule morphology – Nodule diameter	50.29	< 2.2E-16***		15.03	< 2.2E-16***	

Two sub models were made for each of the reconstruction kernel (soft tissue and lung), both at a standardized radiation dose. Abbreviations: APE_volume_: Absolute percentage volumetric error. (Significance codes: ***p < 0.001, **p < 0.01, *p < 0.1, *ns* not significant)

**Fig. 3:**
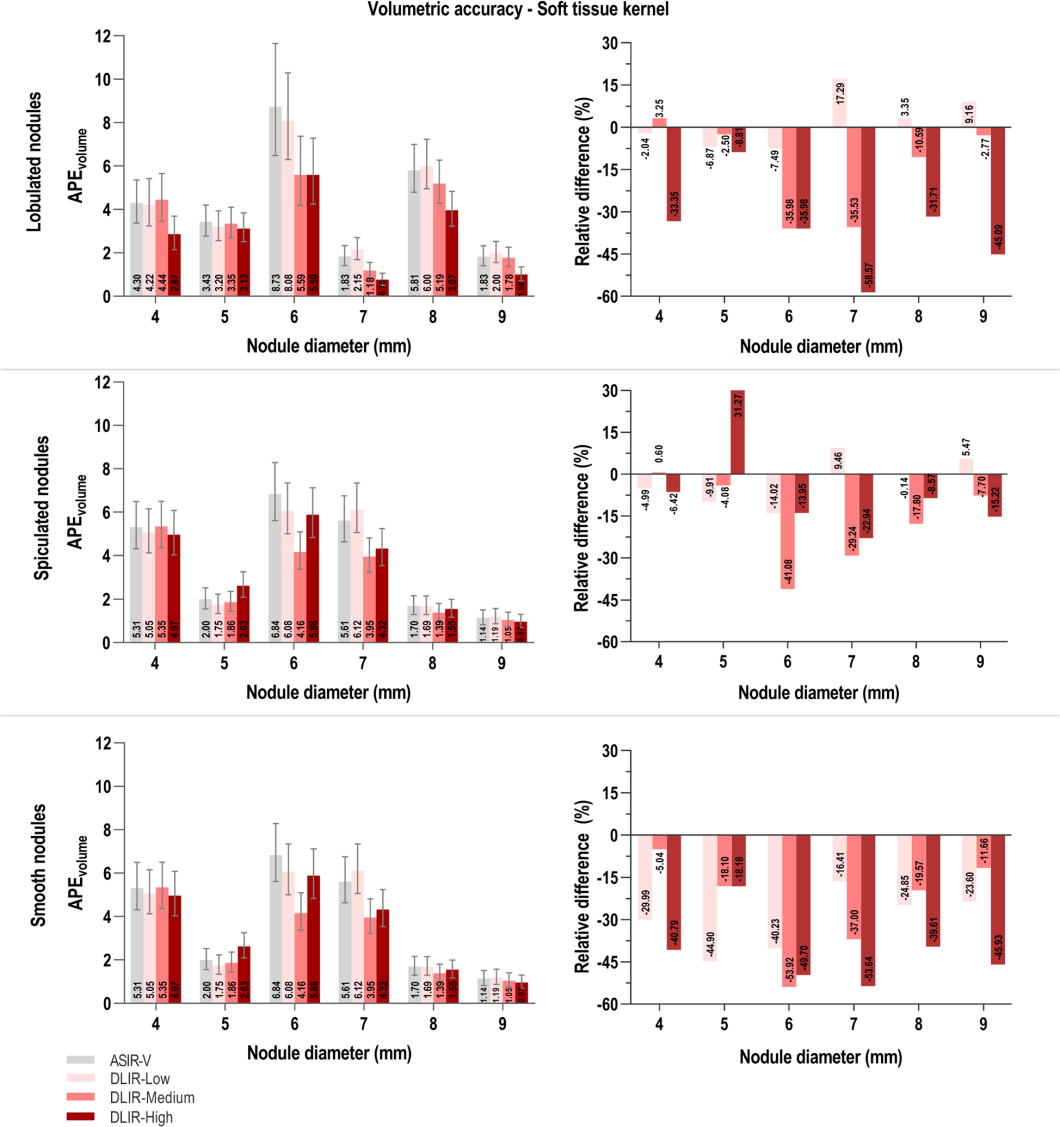
Volumetric error estimates for different nodule morphologies and diameters, at standardized radiation dose with soft kernel. Left: Absolute percentage volumetric error (APE_volume_) for four different reconstruction algorithms (ASIR-V 60%, DLIR-Low, -Medium and -High). Error bars depict the 95% confidence intervals to display the variability on the estimated outcome. Right: Relative difference (%) between APE_volume_ values of ASIR-V compared to different levels of DLIR. Abbreviations: APE_volume_: Absolute percentage volumetric error, ASIR-V: adaptive statistical iterative reconstruction, DLIR: deep learning image reconstruction

Figures 3 (soft tissue kernel) and 4 (lung kernel) depict for a standardized dose, subdivided for the three morphological classes (lobulated, spiculated and smooth nodules) and the six diameter classes (4-9 mm) how different reconstruction algorithms influence volumetric error estimates. In both figures the three graphs on the left show APE_volume_ estimates and three graphs on the right show the relative difference in APE_volume_ when ASIR-V is compared with DLIR at low, medium and high strength.

**Fig. 4:**
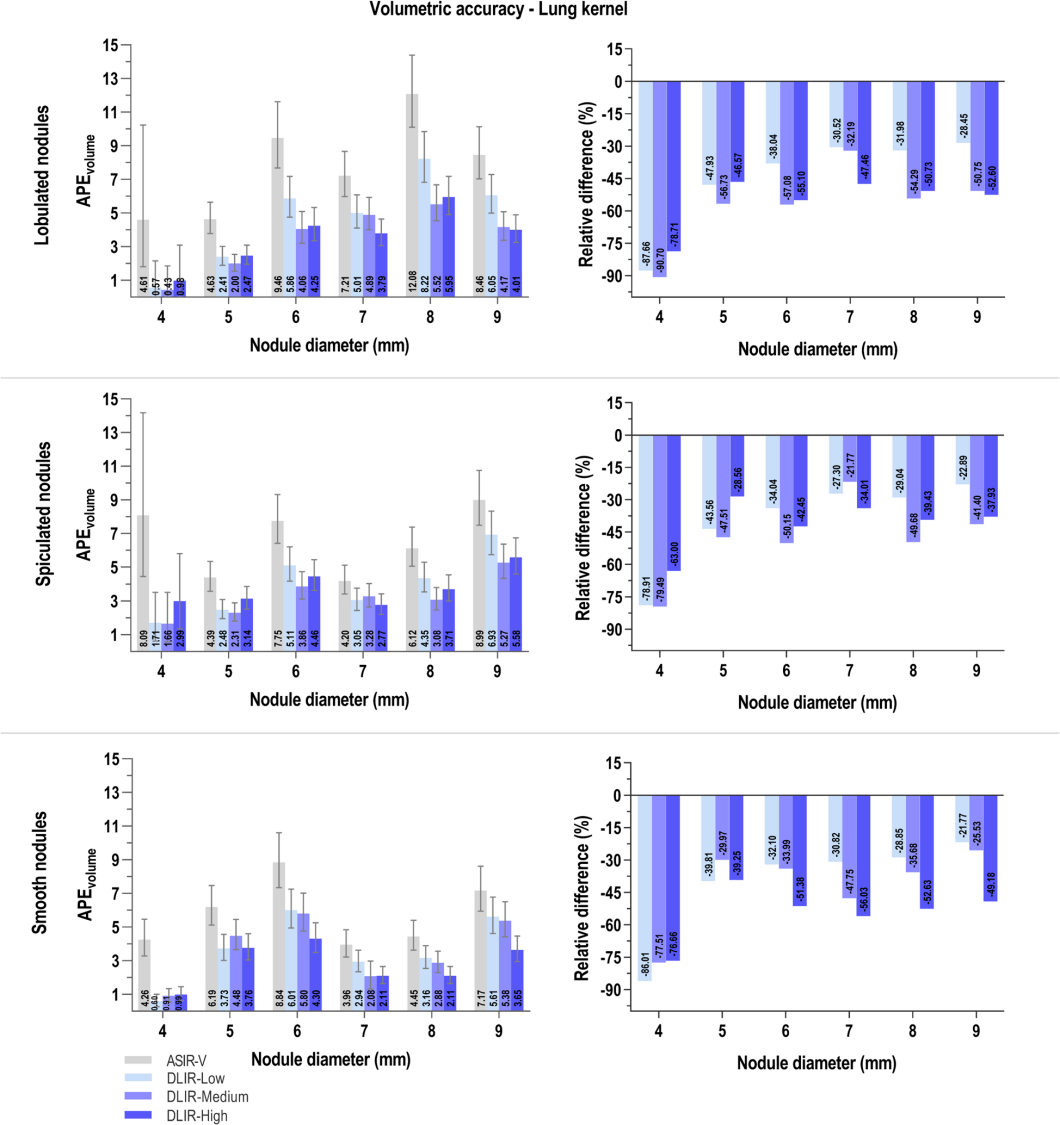
Volumetric error estimates for different nodule morphologies and diameters, at standardized radiation dose with lung kernel. Left: Absolute percentage volumetric error (APE_volume_) for four different reconstruction algorithms (ASIR-V 60%, DLIR-Low, -Medium and -High). Error bars depict the 95% confidence intervals to display the variability on the estimated outcome. Right: relative difference (%) between APE_volume_ values of ASIR-V compared to different levels of DLIR. Abbreviations: APE_volume_: Absolute percentage volumetric error, ASIR-V: adaptive statistical iterative reconstruction, DLIR: deep learning image reconstruction

General observation for both reconstruction kernels is that nodules with smooth margins have lower APE_volume_ values compared to the lobulated and spiculated nodules. Besides, smooth nodules in all diameter classes have overall lower measurement errors when DLIR is applied compared to ASIR-V. This is also visible in relative reduction up to 50% and higher when comparing ASIR-V with DLIR. For the lobulated and spiculated nodules in the soft tissue kernel (Fig. 3), DLIR induces in most cases a reduction in APE_volume_ compared to ASIR-V. However, all three levels of DLIR at different diameters also show some error estimates that are higher than for ASIR-V. APE estimates for the soft tissue kernel (Fig. 3, left) are overall comparatively lower than those for the lung kernel (Fig. 4, left). In all combinations of nodule morphology and diameter for the lung kernel results, DLIR consistently renders lower APE_volume_ estimates and related relative reductions in errors in comparison to ASIR-V (Fig. 4). A general trend that can additionally be seen for the lung kernel results is that especially for the smaller diameters, there is a substantial reduction in volumetric error when applying DLIR.

### Subjective image quality

Exploratory analysis demonstrated that reconstruction kernel is not a significant predictor variable of the outcome variable IQ. Therefore, we did not subdivide results of image quality analysis based on kernel. The predictor variables included in the ordinal logistic regression model and their two-way interaction terms are depicted in Table 4. This table also include the likelihood ratio Chi square statistics and p-values that depict the strength with which predictor variables have an influence on the outcome variable, perceived subjective IQ. All main predictor variables (dose, reconstruction algorithm, morphology and diameter) have a significant influence on the subjective IQ score. For the interaction effects, only the interaction between diameter and reconstruction algorithm showed no significant effect.

**Table 4:** Predictor and outcome variables of the ordinal logistic regression analysis with their according Chi square statistic

**Outcome variable**			
**IQ score**			
**Predictor variable (main effects)**	LR Chi square statistic	*p*-value	
Dose	3390.90	< 2.2E-16***	
Reconstruction algorithm	32.70	3.8E-07***	
Nodule morphology	152.10	< 2.2E-16***	
Nodule diameter	17.00	4.4E-03**	
Predictor variable (interaction effects)			
Dose – Reconstruction algorithm	332.70	< 2.2E-16***	
Dose – Nodule morphology	33.00	2.7E-04***	
Dose – Nodule diameter	60.90	7.8E-05***	
Reconstruction algorithm – Nodule morphology	14.10	2.9E-02*	
Reconstruction algorithm – Nodule diameter	14.00	5.3E-01ns	
Nodule morphology – Nodule diameter	103.30	< 2.2E-16***	
Goodness of fit test	LR statistic	*p*-value	
Lipsitz test for ordinal response models	4.39	0.88ns	

At the bottom of the table, the Lipsitz goodness of fit test for ordinal logistic models is presented. Abbreviations: IQ: image quality, LR: likelihood ratio. (Significance codes: ***p < 0.001, **p < 0.01, *p < 0.1, *ns* not significant)

**Fig. 5:**
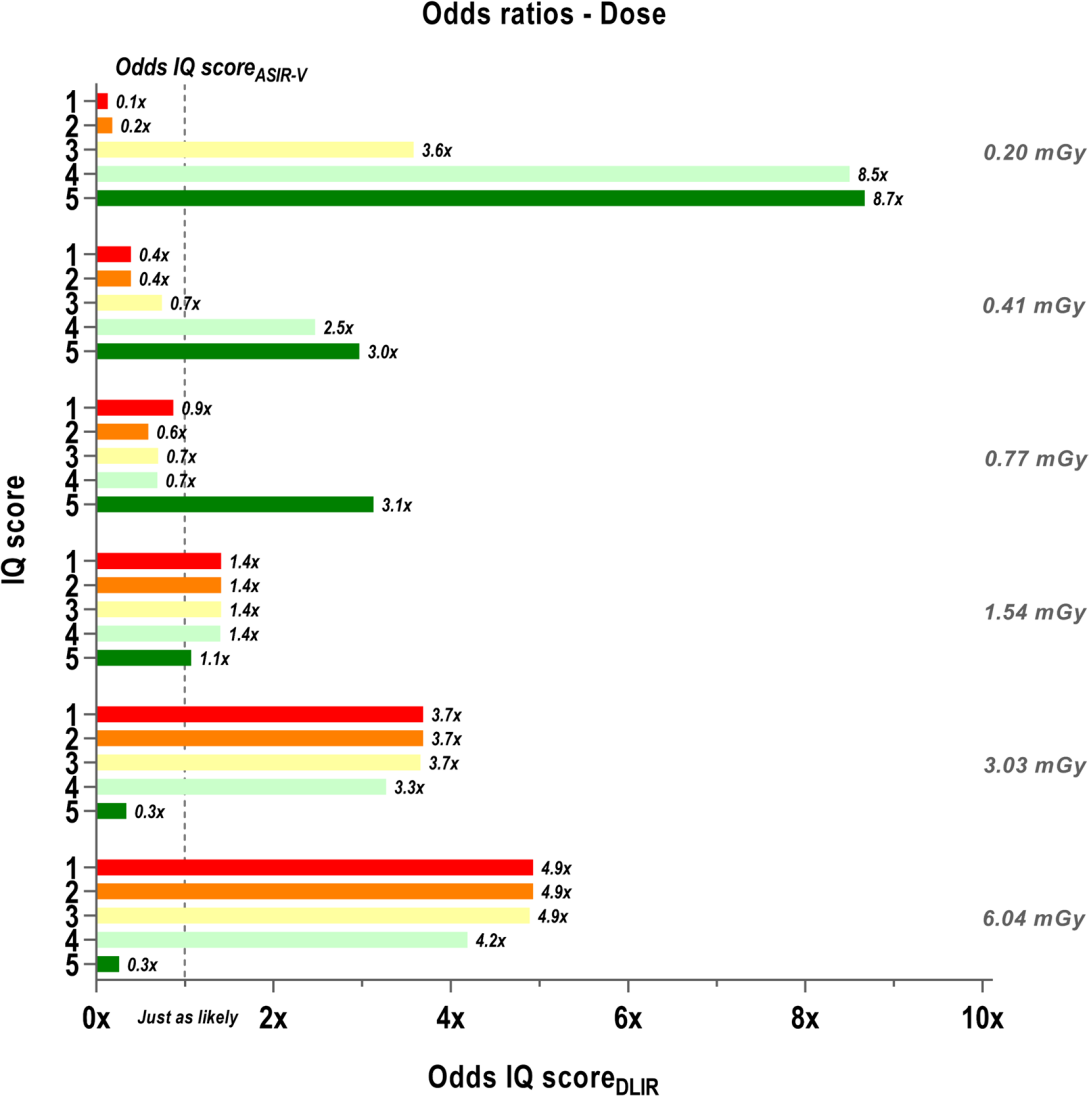
Odds on IQ score with ASIR-V compared to DLIR per radiation dose. Odds ratio (OR) between the odds for ASIR-V 60% to assign an IQ score (Odds IQ score_ASIR-V_) and the odds for DLIR to give the same IQ score (Odds IQ score_DLIR_) grouped by radiation dose. The dotted line indicates where both odds are just as likely to occur for both reconstruction algorithms. Each IQ score (1 to 5) is presented by different color, as depicted by the numbers on the y-axis. Abbreviations: IQ: image quality, ASIR-V: adaptive statistical iterative reconstruction, DLIR: deep learning image reconstruction

**Fig. 6:**
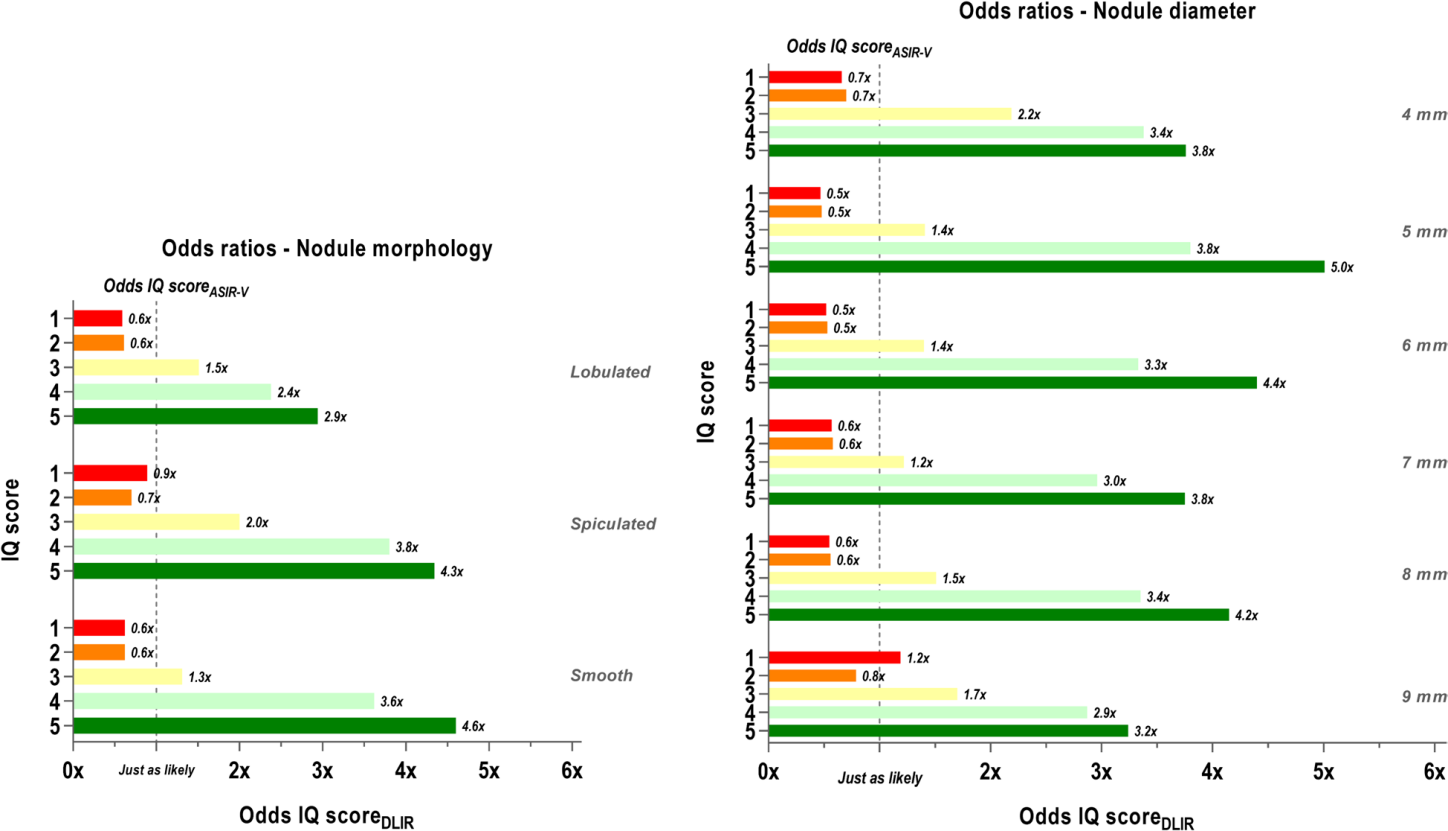
Odds on IQ score with ASIR-V compared to DLIR per nodule morphology and diameter. Odds ratio (OR) between the odds for ASIR-V to ascribe an IQ score (Odds IQ score_ASIR-V_) and the odds for DLIR to give the same IQ score (Odds IQ score_DLIR_) grouped by nodule morphology (left) and nodule diameter class (right). The dotted lines indicate where both odds are just as likely to occur for both reconstruction algorithms. Each IQ score (1 to 5) is presented by different color, as depicted by the numbers on the y-axis. Abbreviations: IQ: image quality, ASIR-V: adaptive statistical iterative reconstruction, DLIR: deep learning image reconstruction

Output of the ordinal logistic regression model is provided as the probability that certain nodule reconstructed with either of the four reconstruction algorithms at certain radiation dose is given a particular IQ score. Computation of odds ratios (OR) from these allows to investigate the potential impact of interchanging ASIR-V for DLIR on the perceived IQ. As such, we can derive how much more (OR > 1) or less (OR < 1) likely radiologists are to assign a particular image score to an image. Figures 5 and 6 display the odds ratios for variation of distinctive variables, respectively dose, morphology and diameter. As there was no additional benefit or difference when looking at the three strength levels of DLIR separately, those three are displayed compared to ASIR-V in a combined manner.

It can be seen on Fig. 5 that for a dose of 1.54 mGy radiologists are about just as likely to give the same IQ score to images reconstructed with ASIR-V and DLIR. For a dose of 0.20 mGy, it is apparent that DLIR on the one hand increases odds to give an IQ score of 3 or higher and on the other hand strongly reduces the odds to give an IQ score of 1 or 2. This is also visible for 0.41 and 0.77 mGy, but less pronounced. In contrast, because of the high odds for an IQ score of 5 with ASIR-V, it is comparatively less likely that the same IQ score will be given to an image with DLIR at 3.03 and 6.04 mGy. Nevertheless, higher odds for DLIR for an IQ score of 4 are detected, but then again the odds for relatively worse IQ scores (≤ 3) are also larger at these radiation doses.

Based on this, and considering the initial approach of this study in relation to LCS, we emphasized further analysis on radiation doses up to 1.54 mGy. Figure 6 left and right show the odds ratios, respectively for nodule morphology and nodule diameter. In these cases, no great discrepancies in the results in general are apparent among the different morphologies and the diameter classes themselves. Nonetheless, this representation shows how DLIR increases the odds to assign the two highest IQ scores (4 and 5) also for the nodules with irregular margins and relatively smaller diameters. Once again, DLIR overall increases odds to give an IQ score higher than 3 while strongly reducing the odds to give an IQ score of 1 or 2.

## Discussion

In the present study, results show that DLIR performs at least as good as the standardly used ASIR-V reconstruction algorithm in terms of volumetric accuracy and subjective IQ in an anthropomorphic chest phantom. DLIR has rendered valuable results for both investigated metrics at the lower radiation doses, which can have potential for low-dose CT LCS programs. Radiation-induced cancers should be considered as a harm and potential risk related to repeated low-dose CT screening [4]. More advanced CT scanners and state-of-the art software must ensure that screening can be conducted at dose levels far below those at the time of the large LCS trials. As such, this dose reduction could be achieved with the implementation of DLIR.

Previous studies have shown that DLIR outperforms conventional reconstruction algorithms in terms of noise, contrast and nodule detection [19], particularly at the lower doses. In our anthropomorphic phantom setting, we found that DLIR resulted in the least error in nodule volume measurements (Fig. 2). Especially in the doses lower than 1 mGy, DLIR outperforms ASIR-V in terms of volumetric accuracy. At the three lowest doses under investigation (0.20, 0.41 and 0.77 mGy) DLIR reduced the percentage error of volume measurements up to 33% for the soft tissue kernel and up to 52% for the lung kernel. At 0.41 and 0.77 mGy for both reconstruction kernels, DLIR-high showed APE_volume_ values that are almost equal to those at the highest dose under investigation (6.04 mGy). Consequently, applying DLIR instead of ASIR-V allows highly accurate nodule volume measurement at greatly reduced CT doses.

Furthermore, DLIR shows a higher perceived subjective IQ at the sub-mGy doses (0.20, 0.41 and 0.77 mGy). Images reconstructed with DLIR are almost 9 times more likely than ASIR-V to render subjective IQ levels as good as high dose images of 11 mGy (Fig. 5). The increased odds of DLIR to provide higher subjective IQ are related to lower noise levels and higher contrast while maintaining a more natural appearance of the images after reconstruction. These results go hand in hand with the improved volumetric accuracy at the lower doses. Several studies have previously reported that DLIR indeed scores better than ASIR-V in terms of objective, task-based image quality characteristics in technical phantoms [12, 15, 20, 31]. As such, the images reconstructed with DL present nodule margins that are less blurred and more distinguishable for the semi-automatic segmentation and volumetry tool. In addition, our results confirm that DLIR has the potential to reconstruct images acquired at ultra-low doses that have a more natural appearance and seem to be preferred by radiologists.

In this study we opted to conduct subjective IQ analysis at various dose levels. Question arises why at the higher doses, such as 6.04 mGy, IQ scores of images reconstructed with DLIR do not remain at the highest level. This phenomenon was also observed in the study of Higaki et al. where they compared noise properties of FBP, two types of IR (hybrid and model based) and DLIR at different radiation doses [29]. The study reported superior IQ in terms of noise properties and spatial resolution for IR at high radiation exposure. Similarly, reduction in dose showed on the other hand improved features for DLIR.

Reconstruction kernel is an image acquisition parameter that also seems to strongly influence volumetric accuracy while we did not see any influence with respect to subjective IQ. It is known that the reconstruction kernel affects the distribution of pixel values and shifts the image noise pattern [17, 40]. With respect to volumetry, it has previously been observed that the segmentation and volumetric accuracy of AI software is affected by sharpness of the kernel [41]. As reported by other studies, the higher the kernel’s value, the sharper the boundary will be between lung nodules and the surrounding lung parenchyma or bronchi [41]. As such, it could be expected that in our set-up with the Lungman phantom, application of the lung kernel accordingly gives rise to sharper edges between lung nodules and air or lung vessels. However, the theoretical improvement of the spatial resolution of a harder kernel occurs at a cost of increasing the noise. In our study, this translates indeed in the fact that absolute volumetric errors, irrespective of the reconstruction algorithm, do lie higher for the lung kernel than for the soft tissue kernel at every radiation dose (Fig. 2). However, the lung kernel in combination with additional DLIR in term resulted in more accurate semi-automatic segmentation and greater reductions in volumetric errors compared to ASIR-V for the same kernel. While DLIR in combination with different kernels improves spatial resolution and as such volumetry, this benefit is not as straightforward for noise properties. The study of Choe et al. demonstrated that assessment and reproducibility of (intra)tumor heterogeneity and texture characterization is highly dependent on the reconstruction kernels with which images were acquired [40]. Therefore, it could be expected that our logistic regression analysis would also show significant influence of reconstruction kernel on the IQ scores. However, this analysis indicated that radiologists in our study did not experience any effect of the reconstruction kernel when scoring subjective IQ. This might be attributable to the fact that all nodules had the same density of a solid nodule and where still mostly spherical. As such, it could be that nodules with different densities and more complex morphologies are more susceptible to the influence of reconstruction kernel on perceived subjective IQ. Nevertheless, our results confirm that the choice of the image acquisition parameter reconstruction kernel potentially influences study results and affects intercomparison and generalizability of different CT acquisitions [40]. The kernel appears to be an important technical parameter of the CT protocol besides the reconstruction algorithm and should therefore be integrated in research questions and study set-ups.

It has been described that DL reconstructed images of perfectly smooth nodules generally show the most accurate volume measurements in phantom studies compared to other reconstruction algorithms [31]. Figures 3 and 4 of this study also show that smooth nodules overall have the lowest APE_volume_ values for all levels of DLIR. Additionally, our study incorporated 3D-printed nodules with lobulated and spiculated margins in order to comprehensively characterize volumetric accuracy and IQ. Nodules without smooth surface and that are relatively smaller in diameter would be expected to give rise to higher inaccuracies in volume measurement and relatively poor quality due to smudged out margins on CT images. Remarkably, our results present that these “more challenging” nodules on images reconstructed with DL actually have volumetric accuracies and subjective IQ which are comparable to or even better than those of smooth nodules of the 9 mm diameter class (Fig. 6). For all levels of DLIR, lobulated and spiculated nodules with diameters of 4 and 5 mm have extreme reductions in APE. In addition, these nodules on DLIR images have higher odds of scoring above average IQ than for the same nodules on ASIR-V images. Hence, extreme dose reduction to sub-mGy levels is also possible for nodules with irregular shapes. This makes DLIR especially interesting for application in LCS CT imaging as it is the purpose of screening to detect nodules as early as possible and distinguish morphological characteristics that could point in the direction of malignancy.

Despite the undeniable advantages of DLIR shown in our standardized anthropomorphic setting, there are several limitations to this study. First of all, AI and DL technologies are still so-called black boxes and phenomena like information loss or hallucinations are never completely ruled out. Even though our study confirms the results of other studies showing a potential added benefit of DLIR [15, 17, 21, 22, 29–31]; one should always take into consideration that DL-based algorithms are not fully understood by the people adapting them. Furthermore, results with regard to DLIR are reported to be vendor specific and they can be influenced by the fact that DL frameworks are either too generic or too finely tuned for specific cases [10, 42]. Since AI-based tools often come with severe expenditures to fully implement in clinical practice, it is fundamental to characterize them comprehensively. Secondly, generalization of results still needs to acknowledge that a phantom was used in this study. The Lungman phantom lacks structures equivalent to the lung parenchyma and lobe fissures. Besides, the low density of the surrounding air is different than that of normal lung tissue in patients. Although our set of 3D-printed nodules included lobulated and spiculated shapes in addition to smooth ones, we realize that these are nonetheless less complex than some morphologies encountered in daily clinical practice. Lastly, even though we corrected for interreader variability in the ordinal logistic regression model, we realize that the analysis of the reproducibility of IQ scoring over time (intrareader variability) also is an important factor to include in future study set-ups***.***

Several future research proposals have emerged in our research group from this study. If DLIR were to get a fundamental role in the performance of LCS, its adaptability and added value in practical use needs to be confirmed. We want to assess the usefulness of DLIR on more image datasets that have different noise levels, that are acquired on other CT scanners and that are reconstructed with DLIR of multiple vendors in an anthropomorphic setting. Besides, computer-aided detection (CAD) tools are increasingly made available by AI companies. These tools are developed to perform nodule detection in combination with nodule volumetry and growth rate calculation, in theory without the interference and initiation of the radiologist [26, 41]. Future studies need to determine whether this fully automated workflow of the software algorithms has the potential to improve the accuracy and to ease clinical work. Lastly, to accommodate to the diversity in which lung cancer can take form in a clinical setting, we want to expand our nodule set. Although nodules with different margins were included, many other morphological characteristics contribute to the assessment of malignancy in clinical practice [23]. These additional features, such as subsolid nodules with ground glass component and more irregular shapes, need to be incorporated to evaluate the impact DLIR has on those features.

## Conclusion

We have observed that DLIR provides promising results that are at least as good as those obtained with the ASIR-V reconstruction algorithm in an anthropomorphic study set-up for low-dose chest CT. Essentially, DLIR allows to achieve notable dose reductions of chest CT onto a level of ultra-low sub-mGy levels while maintaining excellent volumetric accuracy in combination with above average IQ. With the rise of LCS research and implementation, radiation dose reduction has obtained an even more prominent role. DLIR has the potential to keep exposure of participants as low as possible without compromising on volumetric accuracy and IQ, even for lung nodules with small diameters and irregular margins. Besides the reconstruction algorithm, we found that application of different reconstruction kernels substantially influences volumetry on CT images despite being an image acquisition parameter that is often not the focus of research or disregarded in (screening) guidelines and scan protocols. In any case, standardized chest CT protocols, defined by well-considered image acquisition parameters are fundamental for a high-quality lung cancer screening program.

### Supplementary Information

**Additional file 1: Supplementary Table 1.** Standardised β coefficients, with their according standard errors, obtained as output in RStudio from the first multiple linear regression model to investigate the volumetric accuracy for a varying dose. Presented β coefficients were used to calculate the estimates of the mean response, being the absolute percentages volumetric error, which are depicted in Figure 2. Note that the dependent variable is on a logarithmic scale since data follow a log-normal distribution.

**Additional file 2: Supplementary Table 2.** Standardised β coefficients, with their according standard errors, obtained as output in RStudio from the second multiple linear regression model to investigate the volumetric accuracy at a standardized radiation dose for each reconstruction kernel. Presented β coefficients were used to calculate the estimates of the mean response, being the absolute percentages volumetric error, which are depicted in Figures 3 and 4. Note that the dependent variable is on a logarithmic scale since data follow a log-normal distribution.

**Additional file 3: Supplementary Table 3.** Standardised β coefficients, with their according standard errors, obtained as output in RStudio from ordinal logistic regression model to investigate the subjective image quality score. Presented β coefficients were used to calculate the estimates of the odds ratios which are depicted in Figures 5 and 6.

## Data Availability

The datasets generated and/or analyzed during the current study are not (yet) publicly available due to use of datasets in an additional study, but are available from the corresponding author on reasonable request.

## Abbreviations

AI: Artificial intelligence
ALARA: As low as reasonably achievable
APE_volume_: Absolute percentage volumetric error
ASIR-V: Adaptive statistical iterative reconstruction V
BTS: British Thoracic Society
CAD: Computer-aided detection
CT: Computed Tomography
CTDI_vol_: Volumetric Computed Tomography dose index
DLIR: Deep learning image reconstruction
FBP: Filter back projection
IQ: Image quality
IR: Iterative reconstruction
LCS: Lung cancer screening
OR: Odds ratio
TCM: Tube current modulation
