# Supplementary figures and images for: Impact of deep learning image reconstruction on volumetric accuracy and image quality of pulmonary nodules with different morphologies in low-dose CT
Source: Cancer Imaging. 2024 May 9;24:60. doi: 10.1186/s40644-024-00703-w (PMC11080267; doi:10.1186/s40644-024-00703-w)

**Supplementary table 1**


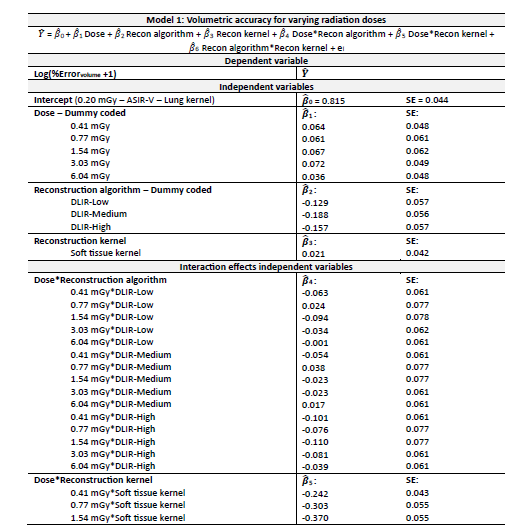

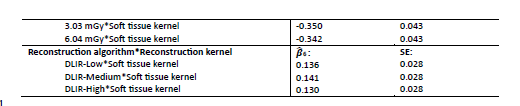

Supplement: Supplementary file 1 — Additional file 1: Supplementary Table 1. Standardised β coefficients, with their according standard errors, obtained as output in RStudio from the first multiple linear regression model to investigate the volumetric accuracy for a varying dose. Presented β coefficients were used to calculate the estimates of the mean response, being the absolute percentages volumetric error, which are depicted in Figure 2. Note that the dependent variable is on a logarithmic scale since data follow a log-normal distribution. [file 40644_2024_703_MOESM1_ESM.docx]

**Supplementary table 2**


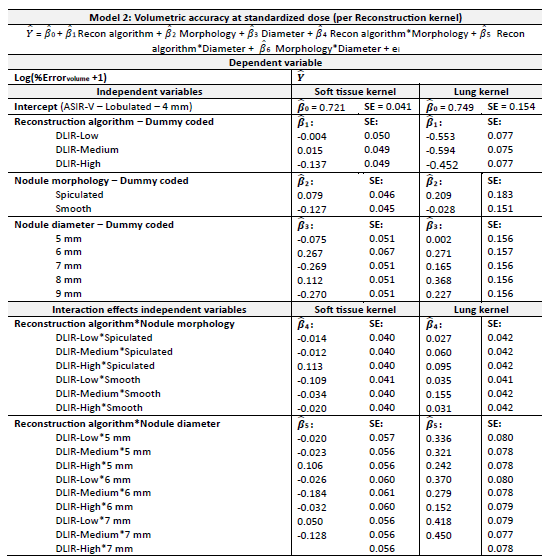

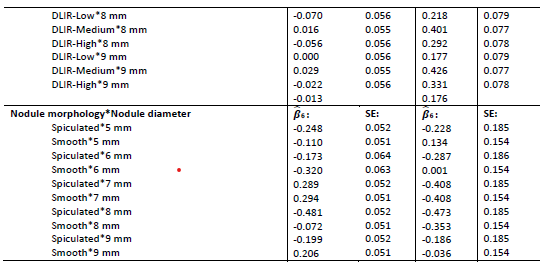

Supplement: Supplementary file 2 — Additional file 2: Supplementary Table 2. Standardised β coefficients, with their according standard errors, obtained as output in RStudio from the second multiple linear regression model to investigate the volumetric accuracy at a standardized radiation dose for each reconstruction kernel. Presented β coefficients were used to calculate the estimates of the mean response, being the absolute percentages volumetric error, which are depicted in Figures 3 and 4. Note that the dependent variable is on a logarithmic scale since data follow a log-normal distribution. [file 40644_2024_703_MOESM2_ESM.docx]

**Supplementary table 3:**


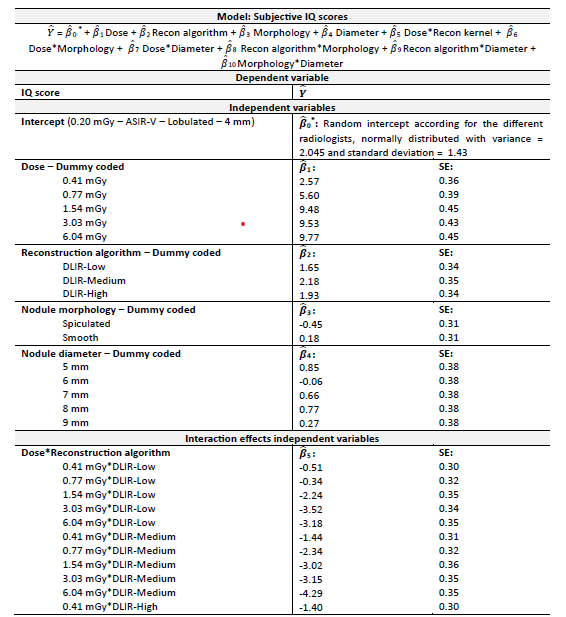

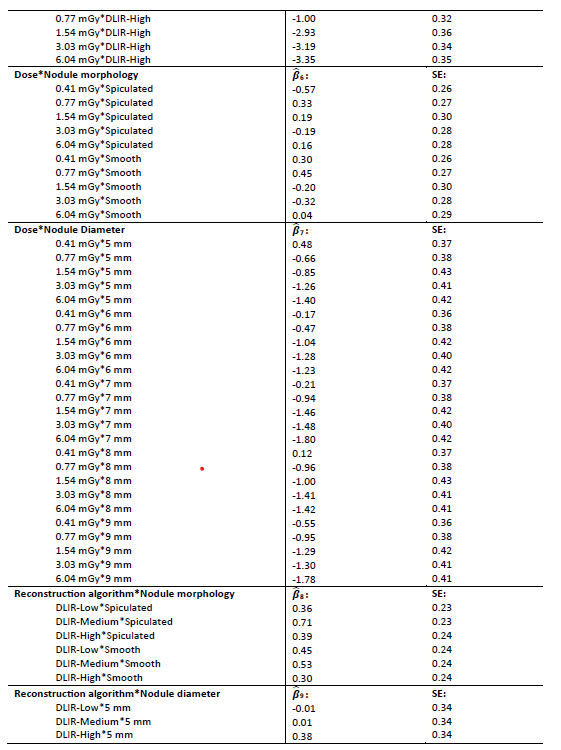

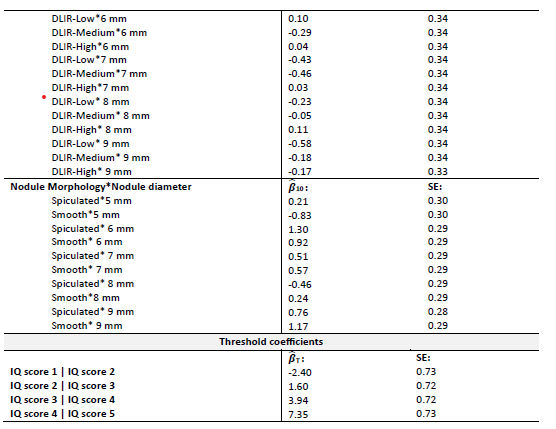

Supplement: Supplementary file 3 — Additional file 3: Supplementary Table 3. Standardised β coefficients, with their according standard errors, obtained as output in RStudio from ordinal logistic regression model to investigate the subjective image quality score. Presented β coefficients were used to calculate the estimates of the odds ratios which are depicted in Figures 5 and 6. [file 40644_2024_703_MOESM3_ESM.docx]
